# Regular Supplementation with Antioxidants Rescues Doxorubicin-Induced Bone Deformities and Mineralization Delay in Zebrafish

**DOI:** 10.3390/nu14234959

**Published:** 2022-11-23

**Authors:** Sunil Poudel, Gil Martins, M. Leonor Cancela, Paulo J. Gavaia

**Affiliations:** 1 Centre of Marine Sciences, University of Algarve, 8005-139 Faro, Portugal; 2 Faculty of Medicine and Biomedical Sciences (FMCB), University of Algarve, 8005-139 Faro, Portugal; 3 PhD Program in Biomedical Sciences, Faculty of Medicine and Biomedical Sciences (FMCB), University of Algarve, 8005-139 Faro, Portugal; 4 Algarve Biomedical Center, University of Algarve, 8005-139 Faro, Portugal

**Keywords:** doxorubicin, MitoTEMPO, oxidative stress, resveratrol, secondary osteoporosis, zebrafish

## Abstract

Osteoporosis is characterized by an abnormal bone structure with low bone mass and degradation of microarchitecture. Oxidative stress induces imbalances in osteoblast and osteoclast activity, leading to bone degradation, a primary cause of secondary osteoporosis. Doxorubicin (DOX) is a widely used chemotherapy drug for treating cancer, known to induce secondary osteoporosis. The mechanism underlying DOX-induced bone loss is still not fully understood, but one of the relevant mechanisms is through a massive accumulation of reactive oxygen and nitrogen species (i.e., ROS and NOS) leading to oxidative stress. We investigated the effects of antioxidants Resveratrol and MitoTEMPO on DOX-induced bone impairment using the zebrafish model. DOX was shown to increase mortality, promote skeletal deformities, induce alterations on intestinal villi, impair growth and mineralization and significantly downregulate osteoblast differentiation markers *osteocalcin 2* and *osterix/sp7*. Lipid peroxidation was significantly increased in DOX-supplemented groups as compared to control and antioxidants, suggesting ROS formation as one of the key factors for DOX-induced bone loss. Furthermore, DOX affected mineral contents, suggesting an altered mineral metabolism. However, upon supplementation with antioxidants, DOX-induced effects on mineral content were rescued. Our data show that supplementation with antioxidants effectively improves the overall growth and mineralization in zebrafish and counteracts DOX-induced bone anomalies.

## 1. Introduction

Osteoporosis is a common metabolic skeletal disorder characterized by abnormal bone structure, low bone mass and degradation of skeleton microarchitecture, leading to bone fragility and increased risk of fracture [1,2]. Oxidative stress induces an imbalance in osteoblast and osteoclast activity, leading to imbalances in bone metabolism, a primary cause of secondary osteoporosis caused by specific medications such as doxorubicin (DOX) [3,4]. Several clinical studies have revealed that antioxidant and/or pro-oxidant mechanisms are involved in bone pathologies such as osteoporosis [5,6,7,8]. DOX has long been recognized among the most toxic anticancer agents, causing large accumulations of reactive oxygen and nitrogen species (i.e., ROS and NOS) that negatively impact bone cell metabolism [9]. NADPH-dependent reductases are capable of producing a one-electron reduction of DOX to DOX-semiquinone free radicals [10,11]. Under aerobic conditions, quinone-semiquinone derived from adriamycin undergoes redox cycling and generates superoxide radicals [12]. Adriamycin free radicals are formed by a non-enzymatic mechanism involving iron. The Fe_2_^+^-DOX free radical complex formed by the redox interaction of adriamycin with Fe_3_^+^ reduces oxygen to hydrogen peroxide and ROS [11,13,14]. This mechanism produces a free radical that induces DNA damage by oxidative injury [15,16] and causes lipid peroxidation [17,18,19,20,21,22] upon DOX exposure. Previously, postmenopausal breast cancer patients under the DOX regimen showed decreased bone mineral density and loss of bone [23]. Similar effects were also observed in DOX-exposed rats [23,24].

Teleost fish, such as the zebrafish (*Danio rerio*), are recognized models for biomedical research, including skeletal development, due to their similarities in molecular mechanisms and signaling pathways with humans [25,26]. In fish, skeletal anomalies are linked with oxidative stress, genetics, epigenetics, and nutritional factors, such as vitamins, minerals, and lipids, which are considered the main influencing nutrients on skeleton development. Antioxidant defense mechanisms of the cells are constantly counteracting ROS produced by endogenous or exogenous sources [27,28] with catalase, superoxide dismutase and glutathione peroxidase acting by scavenging hydrogen peroxide, superoxide and hydroperoxides, respectively [29].

Resveratrol (RES) is a naturally occurring polyphenolic (3,4′,5-trihydroxystilbene) compound found in grapes, cranberries, and nuts [30], with antioxidant, anti-inflammatory, estrogenic, and proliferative properties, which can influence bone metabolism [31]. Previously, it has been shown that RES can counteract glucocorticoid-induced bone damage [32] and zinc oxide-induced oxidative stress [33] in zebrafish. RES has also been shown to improve lipid metabolism homeostasis in zebrafish [34]. MitoTEMPO [MT] is a mitochondria-targeted antioxidant that scavenges mitochondrial superoxide and alkyl radicals [35,36]. MT was shown to reverse tafazzin knockdown-induced mitochondrial ROS production [37]. This suggests MT is a potential compound for counteracting mitochondrial-induced oxidative stress.

DOX treatment has been shown to cause a significant reduction of bone mass in humans [23], mice [24] and gilthead seabream (*Sparus aurata*) [38]. Previously, we have shown the reversal effect of RES and MT over DOX-induced bone impairment on gilthead seabream [38]. We hypothesized that diets supplemented with antioxidants would counteract DOX-induced effects in zebrafish. In this study, taking advantage of the zebrafish as an in vivo model with osteocytic bone, we investigated the effects induced by DOX- on bone and aimed to reverse DOX-induced negative effects by regular supplementation with antioxidants. To the best of our knowledge, no studies have been performed on DOX-induced bone loss in this model. Therefore, this study will further strengthen the previous results obtained in vitro on the effects of DOX, RES and MT on bone development and mineralization and on the reversal of DOX-induced bone impairment by antioxidants.

## 2. Materials and Methods

### 2.1. Housing Conditions

Wild-type zebrafish [AB-strain (ZFIN ID: ZDB-GENO-960809-7)] were maintained at the zebrafish facility of the Centre of Marine Sciences (CCMAR, Faro, Portugal). Adults were crossed to obtain the necessary larvae for this study. The photoperiod of the room was controlled with a 14-h/10-h light/dark cycle, and air humidity was maintained at 60% [39]. Fish were kept in 3.5 L plastic tanks connected to a 980 L recirculating housing system (ZebTEC; Tecniplast, Buguggiate, VA, Italy). Water quality was ensured by a daily water renewal of 10% of total volume in recirculation through an automated pump. Water quality was ensured through filtration: mechanical (pleated cartridge filters, 50 µm), biological (ceramic beads), carbon filter (granular activated), and ultraviolet sterilization (180,000 µWs/cm^2^). The system water temperature (28 °C ± 1 °C), pH (7.5 ± 0.2) and conductivity (750 ± 30 µS/cm) were controlled through an integrated computerized system, and pH and conductivity were maintained stable through the addition of a sodium bicarbonate solution (S5761, Sigma Aldrich, Madrid, Spain) and an Instant Ocean salt concentrated solution (35 g/L; Aquarium systems, Sarrebourg, France), respectively. NO_2_^−^ and NH_4_^+^ values were monitored weekly and maintained < 0.1 mg/L and NO_3_^−^ < 50 mg/L, [40].

### 2.2. Micro Diet Preparation

Microdiets were prepared according to Poudel et al. [38]. Briefly, the microdiets supplemented with antioxidants and pro-oxidants were prepared manually by mixing squid flour, water-soluble components and subsequently with fat and lipid-soluble vitamins, and finally, on warm water with gelatin dissolved. RES (34 mg/kg) (TCI, Tokyo, Japan) [32,38,41] and DOX (30 mg/kg) (TCI) [38,41] were dissolved on polar molecules, whereas MT (5 mg/kg) (Sigma-Aldrich) [38,41,42] was dissolved in water. The dough was first compressed and then made into pellets using a grinder (Severin ZB 5591 Meat mincer, Suderm, Germany). Then the pellets were dried for 24 h at 38 °C in a drying oven Ako, Barcelona, Spain). Finally, dried pellets were crushed and placed through sieves (Filtra Vibración, Barcelona, Spain) in order to obtain varied particle sizes (i.e., 125 µm, 250 µm, and 500 µm) [28].

### 2.3. Feeding Trial

A zebrafish broodstock group of AB strain with 4–5 months, females (n = 20) and males (n = 20), was crossed, and 2500 eggs were collected and incubated at 28 °C ± 0.5 °C in 1 L tanks (Tecniplast) at a density of 200 eggs/L in E2 (embryo medium) with 50 ppt methylene blue (Sigma-Aldrich) to reduce bacterial and fungal growth [43,44]. At 5 days post fertilization (dpf), 2400 larvae were pooled and divided into quadruplicates (100 larvae/L) for each treatment group. The rearing density was gradually decreased every 5 days by increasing the volume of water in the tank, i.e., 5–10 dpf: 100 larvae/L, 10–15 dpf: 66 larvae/L, 15–30 dpf: 33 larvae/L. A volume corresponding to 90% of water was renewed every day with fresh water collected from the zebrafish recirculating system since the trial was conducted in static conditions. The feeding trial was conducted until 30 dpf (post-larvae) when all skeletal structures were predicted to be completely formed [40].

The zebrafish larvae were fed with microdiets supplemented with antioxidant and pro-oxidants alone or combined. The fish were fed three times a day with antioxidant microdiets, whereas pro-oxidant diets were only fed to the fish every 72 h and continued with a combination of control or antioxidant diets. The microdiet combinations were performed by mixed feeding with the pro-oxidant and antioxidant diets. The total amount of diet fed daily per tank was 15 mg and increased 5 mg each week. For the first 2 days, 150 rotifers/mL were added to the experimental tanks during the morning [45]. On the 5th day, microdiet uptake was checked using microphotographic observation [40]. A spatula was prepared with a 3D printer with capacity of 5 mg per scoop to standardize the feeding.

### 2.4. Whole-Mount Double Staining and Evaluation of Skeletal Anomalies

To assess skeletal abnormalities and vertebral mineralization in the post-larvae, whole-mount double staining was performed using an acid-free protocol for bone and cartilage adapted from Gavaia et al. [46] and Walker and Kimmel [47]. A group of 20 post-larvae/replicate were processed for whole mount double staining of the skeleton using alcian blue 8GX (Sigma-Aldrich) for cartilage, and alizarin red S (AR-S) for mineralized structures (Sigma-Aldrich), as described [47]. Briefly, 30 dpf post-larvae were stained with alcian blue solution (0.1% *w/v*) in MgCl_2_ (60 mM) dissolved in 70% ethanol for 3 h followed by rehydration steps for 2 h in a decreasing concentration gradient of ethanol (96% to 25%). Samples were then stained with 0.05% AR-S in 0.5% potassium hydroxide solution (KOH, Sigma-Aldrich) for 16 h. The clearing was performed with 1% KOH and larvae were consecutively transferred through increasing glycerol concentrations (25% to 100%) and stored in 100% glycerol (Merk Millipore, Massachusetts, USA) until examination. Whole mount double stained samples were examined under a stereomicroscope (MZ10F Leica, Wetzlar, Germany). Detection of skeletal anomalies was performed following the nomenclature by Bird et al. [48]. For assessing the mineralization of vertebrae, 20 individuals per group were analyzed. The vertebrae were categorized according to the degree of mineralization as: unmineralized, mineralizing and mineralized according to the intensity of AR-S staining observed, using ImageJ1.53c (Rockville, MD, USA).

### 2.5. Mineral Contents

Samples of zebrafish post-larvae (30 dpf) (N = 8/tank) were dried for 72 h in an oven at 60 °C. Dried and weighted samples were processed with a 65% nitric acid digestion, and microwave (Discover SP-D 80, CEM, Matthews, NC, USA) with magnetic beads for 9 min was used for extraction of minerals. The samples were then diluted in a 1:5 ratio with milli-Q water. Calcium standard (Agilent, Santa Clara, CA, USA), which also contains Fe, Mg, Na and K, and Phosphorus standard (Agilent), were prepared on 5% nitric acid. The mineral contents were measured by microwave plasma-atomic emission spectrometry (MP-AES 4200, Agilent, Santa Clara, CA, USA) at 393.366 and 213.318 nm wavelength for Calcium and Phosphorus, respectively. The intensity values obtained from the samples were compared with the standard curve.

### 2.6. Lipid Peroxidation (MDA) Analysis

Lipid peroxidation was measured using the malondialdehyde (MDA) assay kit (Sigma-Aldrich) by reacting MDA with thiobarbituric acid substance (TBARS). Approximately 25–30 mg of larval sample was homogenized on 20% trichloroacetic acid (*w/v*) (1.5 mL) with 0.05 mL of 1% BHT in methanol. To the primary solution, 2.95 mL of 50 mM thiobarbituric acid was added, then mixed and heated for 10 min at 100 °C. The protein precipitates were extracted by centrifugation at 2000× *g*, and the absorbance was measured using the Evolution 300 spectrophotometer (Thermo Scientific, Loughborough, UK) at 532 nm. The MDA standard curve was plotted, and the absorbance of samples was compared against the standard. TBA-MDA concentration was expressed as nmol MDA/mg of tissue [49].

### 2.7. RNA Extraction and qPCR

NZYol Reagent (NZYtech, Lisbon, Portugal) was used to extract total RNA from 10 whole specimens at 30 dpf. DNase I treatment (Promega, Madison, WI, USA) was performed with 1 µg RNA for 30 min at 37 °C and the RNA was reverse-transcribed at 37 °C for one hour using M-MLV reverse transcriptase (Invitrogen, Waltham, MA, USA), RNaseOUT (Invitrogen) and oligo-d(T) primer [5′-ACGCGTCGACCTCGAGATCGATG(T)13-3′]. qPCR assays were carried out using a Bio-Rad CFX thermocycler (Bio-RAD, Hercules, CA, USA). Gene expression levels were normalized using *eef1a1l1* as a housekeeping gene [50], and the ∆∆Ct method was applied to determine relative quantification [38,51]. The sequence of primers used in this study is listed in Table 1.

### 2.8. Histology

Sample preparation and tissue processing for the histological protocol were performed as described in Cardif et al. [52]. Prior to paraffin inclusion, decalcification was performed with 10% EDTA and 1% PFA. 5 μm tissue sections were prepared using rotary microtome Microm HM 340 (Microm International GmbH, Walldorf, Germany). Slides were then stained with hematoxylin and eosin, as described by Fischer et al. [53]. Blind evaluation of histological parameters was performed to analyze intestinal *villi* length [54,55]. VisiCam 3 Plus (Avantor VWR, Radnor, PA, USA) was used to capture the images from a standard light microscope (Zeiss, Dresden, Germany), and length of *villi* was measured using ImageJ1.53c software.

### 2.9. Statistical Analysis

The data obtained from the skeletal anomalies analysis followed the nomenclature adapted from Gavaia et al. [46] and Walker and Kimmel [47] and was coded according to the typology of the deformities. The data for the mineralization of the vertebrae were coded as mineralized, mineralizing, and unmineralized and then the cumulative percentage was calculated. Partial Least-Squares Discriminant Analysis (PLS-DA) was analyzed using the MetaboAnalystR 3.0 R package and MetaboAnalyst [56,57]. The significance of class discrimination was verified by performing a permutation test (*p* < 0.001; 0/1000), and the performance was measured using the “B/W ratio” as proposed by Bijlsma et al. [58]. MetaboAnalystR 3.0 R package and MetaboAnalyst 5.0 was used to analyze univariate and multivariate analysis [56,57]. Normal (Gaussian) distribution of the data was analyzed using the obtained data by the Anderson–Darling test, D’Agostino and Pearson test, Shapiro–Wilk test and Kolmogorov–Smirnov test. For the analysis of outliers ROUT (Q = 1%) test was performed. Level of significance was analyzed using Student’s *t*-test, One-way ANOVA and Two-way ANOVA on Graphpad prism 8 and IBM SPSS 16. Bar graphs are presented as mean ± SEM. Homogeneity of variance was analyzed with Levene’s test. Differences in *p*-value ≤ 0.05 were considered significant (ns—*p* > 0.05, *—*p* ≤ 0.05, **—*p* ≤ 0.01, ***—*p* ≤ 0.001, ****—*p* ≤ 0.0001).

## 3. Results

### 3.1. Fish Growth and Survival

**Figure 1:**
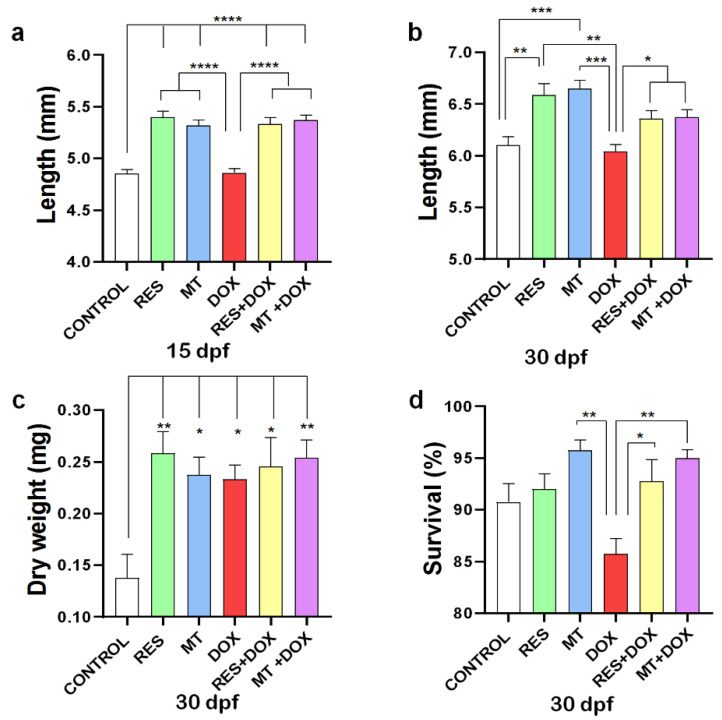
Growth and survival. Larvae were fed with resveratrol (RES), MitoTEMPO (MT) and doxorubicin (DOX) alone or in combination for 30 days. Total length of zebrafish larvae at 15 days post fertilization (dpf) (N = 25 × 4) (**a**) and 30 dpf (N = 25 × 4) (**b**), Dry weight at 30 dpf (N = 10 × 4) (**c**) and Survival at 30 dpf (N = 100 × 4) (**d**). Levels of significance were calculated using Tukey’s multiple comparisons (one-way ANOVA) [* *p* ≤ 0.05, ** *p* ≤ 0.01, *** *p* ≤ 0.001, **** *p* ≤ 0.0001].

The antioxidant supplementation (RES, MT) on microdiets showed to significantly increase the fish standard length at both 15 and 30 dpf, as compared to control and DOX. However, no significant differences in length were observed between DOX and control at 15 and 30 dpf (Figure 1a,b). However, while combining DOX with RES or MT, both antioxidants significantly increased the standard length of the larvae at both time points. No significant differences were observed in dry weight between the groups supplemented with RES, MT and DOX alone or in combination. However, a significant increment in body weight was observed between the control and all other microdiet-supplemented groups at 30 dpf (RES, MT, DOX, DOX + RES, DOX + MT) (Figure 1c). Although the DOX diet significantly reduced the survival of larvae at 30 dpf as compared with MT, the DOX-induced mortality of the larvae was significantly rescued by antioxidant supplementation (Figure 1d). Moreover, the microdiets prepared manually were compared with the commercially available standard diet for zebrafish (ZEBRAFEED, Sparos Lda, Olhão, Portugal), and no significant differences were observed in growth performance (Supplementary Figure S1a).

### 3.2. Intestinal Villi Morphology on Antioxidant and Pro-Oxidant Supplemented Groups

**Figure 2:**
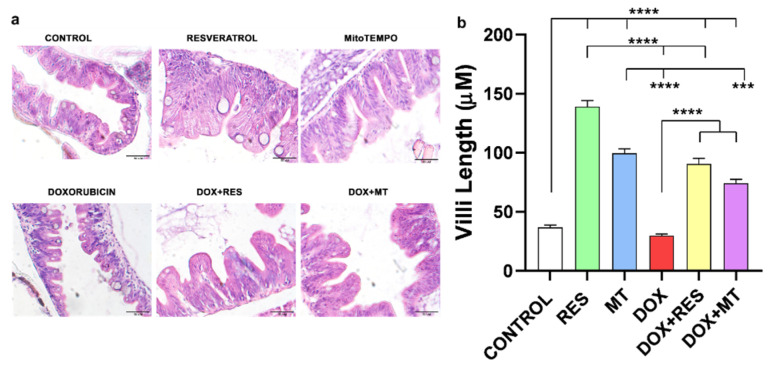
Histology of zebrafish gut. Total of 30 dpf zebrafish guts sections was stained with H&E to observe the villi from the different treatment groups with resveratrol (RES), MitoTEMPO (MT) and doxorubicin (DOX) (**a**). Length of villi (**b**). Statistical significance was calculated using Tukey’s multiple comparison (one-way ANOVA) [*** *p* ≤ 0.001, **** *p* ≤ 0.0001] [N = 5 × 4].

The histological sections were stained with hematoxylin and eosin (H&E) (Figure 2a), and villi length was measured (Figure 2b). Histological examination revealed that regular supplementation of antioxidants RES and MT significantly increased the villi length as compared to DOX and control. Similarly, supplementation of antioxidants combined with DOX significantly increased the length of villi compared to DOX alone (Figure 2a,b). Therefore, supplementation of antioxidants (RES and MT) significantly protects against the DOX-induced negative effects on the intestinal mucosa of zebrafish.

### 3.3. Antioxidants Prevent DOX-Induced Skeletal Deformities

**Figure 3:**
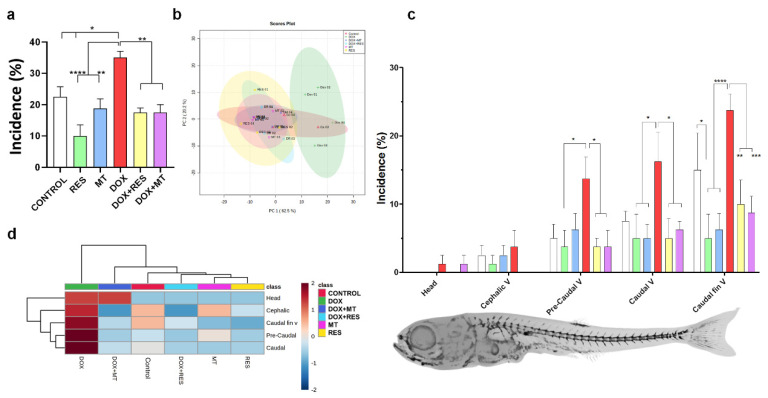
Incidence and distribution of skeletal deformities. Incidence of skeletal deformities (**a**). Partial Least-Squares Discriminant Analysis (PLS-DA score) on the incidence of deformities of zebrafish supplemented with antioxidants, resveratrol (RES), MitoTEMPO (MT), and pro-oxidant, doxorubicin (DOX) (**b**). Distribution of skeletal deformities (**c**) and heatmap of distribution of skeletal deformities (**d**). Statistical significances were calculated using Tukey’s multiple comparisons (one-way ANOVA) [* *p* ≤ 0.05, ** *p* ≤ 0.01, *** *p* ≤ 0.001, **** *p* ≤ 0.0001] [N = 20 × 4].

Skeletal deformities were analyzed at 30 dpf. RES supplementation significantly reduced the incidence of skeletal deformities as compared to the control. In contrast, MT did not show any significant difference in incidence compared to the control. As expected, DOX showed a significantly increased incidence of skeletal deformities compared to antioxidant-supplemented groups and control groups. However, in the fish fed a combination of DOX with antioxidants (RES or MT), a significant reduction in the incidence of deformities was observed compared to DOX treatment (Figure 3a).

Partial Least-Squares Discriminant Analysis (PLS-DA score) [58] was used to examine the discriminant and similarities in the incidence of skeletal deformities on zebrafish at 30 dpf treated with RES, MT and DOX alone or in combination (Figure 3b). The cluster analysis showed that the DOX-supplemented group was distinct and completely separated from other groups, while [control, antioxidants (RES and MT) and combination of antioxidants and DOX] formed a grouping cluster (Figure 3b). The region-specific skeletal deformities between the microdiet-supplemented groups are presented in Figure 3c. A higher incidence of skeletal anomalies was observed in the caudal fin vertebrae region, followed by the caudal vertebrae and pre-caudal regions. A low incidence of deformities was observed on the head as compared to the axial skeleton. DOX supplementation showed a significantly increased incidence of axial skeleton deformities compared to RES and MT, while combining DOX with antioxidants RES and MT significantly rescued DOX-induced deformities on the axial skeleton. The heatmap depicts the distribution of skeletal deformities, where the DOX-supplemented group was completely different from other groups (Figure 3d). The red color indicates the high incidence of deformities observed on individuals supplemented with DOX, whereas zebrafish supplemented with RES and MT showed lower incidence intensity, as indicated by the blue color. The most common skeletal deformities observed in this study were vertebral fusions, compressions, lordosis, scoliosis, and shortened vertebrae (Supplementary Figure S1b).

### 3.4. Antioxidants Improve Mineralization of the Axial Skeleton

**Figure 4:**
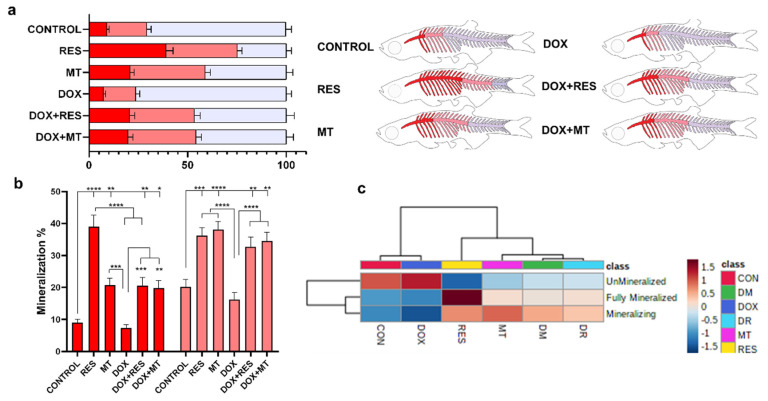
Mineralization of zebrafish vertebral column. Percentage of mineralized (red), mineralizing (pink), unmineralized (blue) vertebrae (**a**) and graphical representation of the mineralization status of the zebrafish. Mineralization status of the vertebrate (**b**), (red represents mineralized vertebrae and pink represents mineralizing vertebrate). Heat map of mineralization of vertebrae of zebrafish feed with resveratrol (RES), MitoTEMPO (MT) and doxorubicin (DOX) supplemented microdiets (**c**). Levels of significance were calculated using Tukey’s multiple comparisons (one-way ANOVA) [* *p* ≤ 0.05, ** *p* ≤ 0.01, *** *p* ≤ 0.001, **** *p* ≤ 0.0001] [N = 20 × 4].

To investigate the effect of the tested antioxidants and pro-oxidant on mineralization of the axial skeleton, whole-mount double-stained larvae were analyzed for mineralization patterns and malformations of the skeleton. The mineralization pattern was categorized as fully mineralized, mineralizing and unmineralized, according to the intensity of the AR-S stain. The RES-supplemented group showed a significantly increased mineralization of the vertebrae (fully mineralized—38% and mineralizing—37%) as compared to control (fully mineralized—9% and mineralizing—20%) and DOX (fully mineralized—7% and mineralizing—16%). The MT treatment also significantly increased the mineralization of vertebrae (fully mineralized—20% and mineralizing—38%) compared to control and DOX groups (Figure 4a,b). The extent of fully mineralized vertebrae increased significantly upon RES supplementation compared to MT. Similarly, while combining DOX with antioxidants, the mineralization of the vertebrae was significantly increased [DOX + RES (fully mineralized—20% and mineralizing—33%), DOX + MT (fully mineralized—15% and mineralizing—37%)] as compared to DOX alone (Figure 4a,b). The heatmap depicts the mineralization pattern of the axial skeleton among the microdiets supplemented groups. The clustering analysis on the heatmap shows that mineralization of the vertebrae is categorized into two distinct groups: control, DOX and RES, MT, DOX + RES, DOX + MT. Control and DOX groups were different from the remaining, displaying lower mineralization of vertebral bodies. Similarly, on the second cluster, RES also showed a distinct mineralization pattern, with stronger mineralization as compared to MT, DOX + RES and DOX + MT (Figure 4c).

### 3.5. Doxorubicin Affects Mineral Content

**Figure 5:**
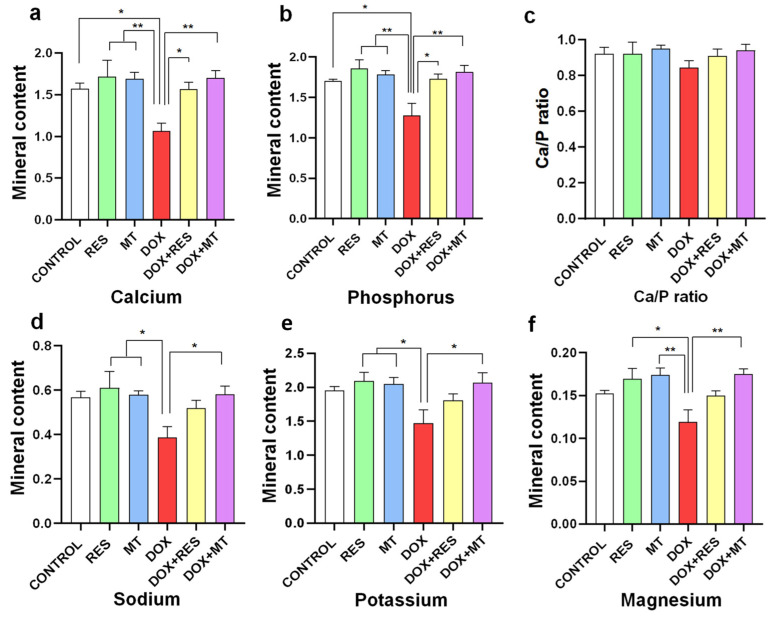
Mineral analysis of zebrafish fed with microdiets supplemented with antioxidants, resveratrol (RES) and MitoTEMPO (MT), and pro-oxidant, doxorubicin (DOX). Mineral content of Calcium (**a**), Phosphorus (**b**), Ca/P ratio (**c**), Sodium (**d**), Potassium (**e**) and Magnesium (**f**). Level of significance was calculated using Tukey’s multiple comparisons (one-way ANOVA) [* *p* ≤ 0.05 and ** *p* ≤ 0.01] [N = 8 × 4].

DOX supplementation significantly reduced contents in calcium (Figure 5a), phosphorus (Figure 5b), sodium (Figure 5d), potassium (Figure 5e) and magnesium (Figure 5f) compared to the antioxidant (RES and MT) treated groups. However, the calcium/phosphorus ratio (Figure 5c) was not significantly altered between the groups since both minerals varied to comparable extents. While combining DOX with antioxidants (DOX + RES and DOX + MT), the calcium and phosphorus content was increased significantly as compared to DOX alone. Similarly, sodium (Figure 5d), potassium (Figure 5e) and magnesium (Figure 5f) contents were significantly increased while co-supplementing DOX and MT.

### 3.6. Antioxidants Reverse Doxorubicin-Induced Oxidative Stress

**Figure 6:**
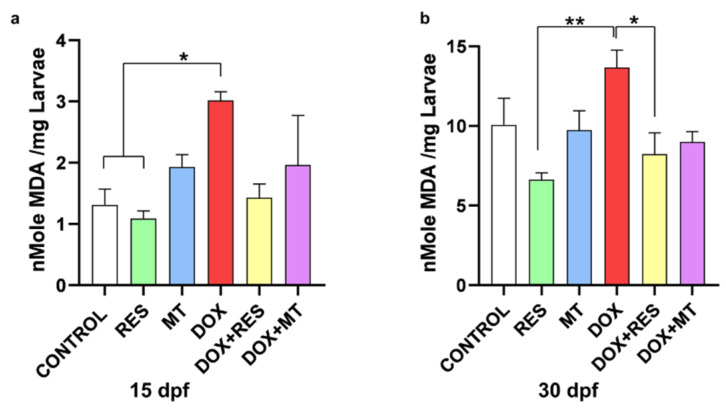
Lipid peroxidation on zebrafish fed microdiets enriched with antioxidants, resveratrol (RES) and MitoTEMPO (MT), and pro-oxidant, doxorubicin (DOX). Lipid peroxidation of the zebrafish larvae at 15 dpf (**a**) and 30 dpf (**b**). Levels of significance were calculated using Tukey’s multiple comparisons (one-way ANOVA) [* *p* ≤ 0.05 and ** *p* ≤ 0.01].

Malondialdehyde (MDA) is the end product of lipid peroxidation, commonly used as a marker for assessing oxidative damage due to the increase of free radicals. The MDA level was significantly higher in the DOX-supplemented group compared to RES and control at 15 dpf (Figure 6a). Similarly, at 30 dpf, the lipid peroxidation was significantly increased in DOX-supplemented groups compared to RES and DOX + RES (Figure 6b).

### 3.7. Doxorubicin-Induced Effects on Osteoblast Differentiation Markers

**Figure 7:**
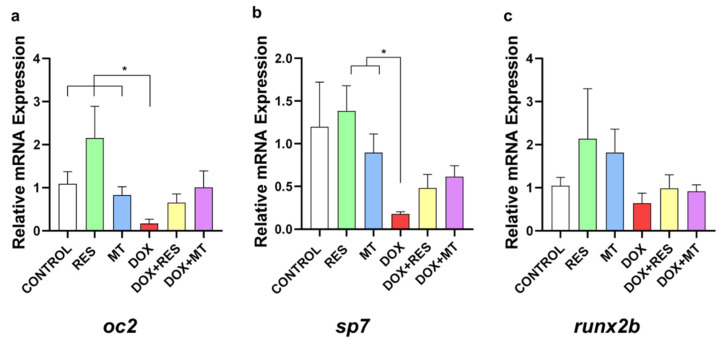
Doxorubicin affects osteoblastic markers. mRNA expression of osteoblast differentiation markers on zebrafish fed with resveratrol (RES), MitoTEMPO (MT) and doxorubicin (DOX) supplemented microdiets, alone or in combination; *osteocalcin* 2 (*oc2*) (**a**), *osterix/sp7 (sp7)* (**b**) and *runx2b* (**c**). Levels of significance were calculated using Student’s *t*-test [* *p* ≤ 0.05] [N = 10 × 4].

In the group supplemented with DOX, it was observed a significant reduction in osteoblastic differentiation marker mRNAs, including the mature osteoblast marker *osteocalcin 2 (oc2) (*Figure 7a) and the immature osteoblast marker *osterix/sp7 (sp7)* (Figure 7b) as compared to RES and MT. However, no significant differences were observed in the mRNA expression of early differentiation marker *runx2b (*Figure 7c).

## 4. Discussion

Resveratrol (RES) was previously shown to have antioxidant, anti-inflammatory, estrogenic-like and cell proliferative properties [30,31]. Several in vivo and in vitro studies have investigated the effect of RES on bone differentiation and remodeling [30,31,32,33]. In the zebrafish model, RES was shown to protect against glucocorticoid-induced bone damage [32] and zinc oxide-induced oxidative stress [33] and also improved lipid metabolism homeostasis in zebrafish [34]. Previously, doxorubicin (DOX) has also been shown to increase systematic bone loss and reduce osteoblast differentiation [23,24,59]. Furthermore, during DOX treatment in patients, an increased risk of bone metastasis and osteolytic injury has been reported [60,61].

In our study, it was demonstrated that RES positively affected growth, with fish presenting a significantly increased length as compared to the control and the group supplemented with the pro-oxidant DOX. Dietary RES supplementation also significantly increased the standard length of zebrafish larvae compared to commercially available standard diets (ZEBRAFEED, Supplementary Figure S1a). DOX has been known to be a highly toxic anticancer drug [62]. DOX-induced developmental toxicity has been studied on various animal models such as dogs [63], rats [64] and zebrafish [65,66,67]. Chang et al. [67] previously reported DOX-induced developmental toxicity on zebrafish, where fish subjected to higher concentrations of DOX (≥25 mg/L) showed acute lethal effects, while fish on lower concentrations (≤0.1 mg/L) showed sublethal effects as well as multiple malformations [67]. Our results revealed no significant differences in standard length between the DOX-supplemented group and the control; however, the standard length was significantly decreased compared to RES and MitoTEMPO (MT). Furthermore, the survival of the larvae was adversely affected by DOX, whereas co-supplementation of RES and MT had a protective effect over DOX-induced mortality. Several other studies have also indicated that, in agreement with our observations, the antioxidants RES [32,68,69] and MT [70] significantly improve overall health and promote the growth of both mammalian and fish models. Retardation in growth is also considered a marker of chronic stress [71], where antioxidants (RES and MT) have been shown to confer protection against these effects.

Metabolism and absorption of dietary nutrients occur in the jejunum. The jejunum is responsible for absorbing most of the nutrients, such as carbohydrates, fats, minerals, proteins and vitamins. The intestinal villi increase the surface area for food absorption and add digestive secretions. In this study, we have shown that antioxidant supplementation significantly increased the length of intestinal villi, which contributes to higher nutrient absorption resulting in enhanced fish growth. Several reports have pointed out that DOX administration caused severe damage to the intestine by increasing apoptosis of jejunal epithelium [72], increased influx of leukocytes and reduced villi length [73], which was also observed in our study. In addition to that, in this study, antioxidants (RES and MT) induced an increase in the villi length and conferred protection against DOX-induced damage on the intestinal villi of larval and juvenile zebrafish. Zhou et al. [74] have reported similar findings in pigs, showing that antioxidants protect against free radical-induced intestinal injury and counteract oxidative stress by modulating p53 mRNA expression. In our study, the negative effects on intestinal villi are suggested as an explanation for the growth retardation of the larvae fed with DOX-supplemented microdiets.

As previously described, higher concentrations of DOX showed acute lethal effects, while lower concentrations (≤0.1 mg/L) showed sublethal effects, such as developing multiple malformations [75]. The concentration of DOX used in this study was 30 mg/kg of diet, which is high compared to previous studies performing oral administration of DOX (10 mg/kg orally) on mice [75]. According to pharmacokinetics analysis, the maximum concentration (Cmax) and maximum time (Tmax) of plasma DOX concentration were 0.2062 µL/mL and 2 h, respectively [75]. Considering that the leaching of micronutrients from the diet in the aquatic environment is 30–35 percent [76,77], the amount of DOX supplemented on the microdiets (30 mg/kg) is sufficient for an effective concentration after the leaching.

The dietary supplementation with DOX at 30 mg/kg that we have used induced an increase in larval mortality by 15%, which is significantly different from other groups. Moreover, this concentration showed a significantly higher incidence of skeletal deformities as compared to the other experimental groups. In contrast, co-supplementation with the antioxidants RES and MT was able to rescue the adverse effects of DOX and reduce the incidence of deformities, increasing survival and mineralization. In this study, the incidence of skeletal anomalies was more concentrated on caudal vertebrae and on the caudal fin vertebrae regions, which is in agreement with previous findings in zebrafish [40] and other aquacultured species (i.e., *Sparus aurata*) [38,78,79,80,81,82,83]. The deformities in the caudal region can lead to secondary vertebral deformities as a result of insipid swimming behavior that affects the growth and conversion index of the fish [79,84]. Therefore, our data confirm the hypothesis and indicate that RES and MT supplementation in the diet would be beneficial for counteracting the DOX-induced bone deformities and for the overall development of the fish.

Mineralization and differentiation of the bone fully depend upon the osteoblast population, which is tightly regulated by osteocytes [85]. In our study, the larvae supplemented with DOX have shown decreased mineralization of the vertebrae compared to groups fed antioxidants (RES and MT). Development and mineralization of the axial skeleton on zebrafish start from the calcified centra of the Weberian region and are followed by rays of the caudal fin simultaneously [48,86]. Here, the effect of antioxidants on the mineralization of the vertebrae is expected to be due to increased osteoblast proliferation and differentiation.

The other factor responsible for the mineralization of bone is mineral metabolism [87]. Bone is the main calcium and phosphate reservoir in higher vertebrates [88]. However, fish absorb different mineral elements from the medium since water contains abundant calcium. Therefore, calcium deficiency is uncommon in fish. However, the only source of phosphorus is food, where a reduction in phosphorus results in low bone mineralization, development of skeletal abnormalities and reduced growth [89,90]. The significant reduction of calcium, phosphorus, sodium potassium and magnesium in the DOX-supplemented groups indicates that DOX alter overall mineral metabolism. Calcium and phosphorus are associated with bone mineralization; the inorganic phase of the bone is composed of calcium phosphates predominantly as hydroxyapatite [Ca_10_(PO_4_)_6_(OH)_2_] [91]. Calcium homeostasis maintains the absorption of calcium and phosphorus from the intestine and maintains levels in bone [91]. In addition to bone metabolism, phosphorus as phosphate (HPO_4_^2−^) is a crucial signaling molecule, an important component of the cell wall, and is essential for RNA and DNA structure, and termed as the currency of energy metabolism as (ATP, ADP and AMP) [87,88,89,90,91]. The lower calcium and phosphorus content suggests that overall bone metabolism is affected by DOX. Therefore, based on our results, we hypothesize that reduced capacity for mineral absorption leads to the lower bone mineral content observed, resulting in lower mineral deposition and contributing to the increased incidence of skeletal deformities observed in DOX-supplemented zebrafish larvae.

An increase in ROS is one of the working mechanisms of action of DOX-induced toxicity [9,61,62,92]. The increased ROS production results in lipid peroxidation, which is a crucial mechanism for DOX-induced toxicity [17,18,19,20,21,22]. Under normal conditions, ROS produced by the cell is balanced by the antioxidant defense mechanism of the cells [29,93]. Here, when zebrafish was supplemented with DOX alone or in combination for 30 days, MDA concentration was significantly increased on DOX supplementation, but co-supplementation with RES and MT could prevent this effect on lipid peroxidation, in accordance with previously reported results [17,18,19,20,21,22]. This signifies that antioxidant supplementation on feed would protect against ROS-induced oxidative stress and lipid peroxidase [94,95].

Osteocalcin (Bglap or osteocalcin, Oc2) is a secreted non-collagenous matrix protein essential in skeletal development and calcium metabolism. Oc2 is a Ca2^+^-binding vitamin K-dependent protein produced by osteoblasts, essential for the differentiation and mineralization of the extracellular matrix [96]. Osterix/Sp7 is a zinc finger transcription factor crucial for osteoblastogenesis during skeletal development [97]. Similarly to the mammalian osteoblast differentiation transcription factors, Runx2 and Sp7 were demonstrated to regulate osteoblastic differentiation during zebrafish bone formation [98]. The significant decrease in the osteoblast differentiation markers induced in the DOX supplementation groups indicates that DOX impairs bone formation and mineralization processes in zebrafish. Oc2 plays an essential role in bone mineralization due to its ability to bind with high-affinity bone hydroxyapatite [99] and Sp7 is essential for early osteoblast differentiation [98]; the mRNA expression of *oc2* and *sp7* was downregulated by DOX, which correlates with the lower mineralization of the vertebrae observed in zebrafish at 30 dpf.

Our data provide evidence that regular supplementation with antioxidants could rescue DOX-induced bone deformities and mineralization in zebrafish. However, some of the limitations of this study should be considered. Firstly, the concentration provided on the microdiets has partially leached in the aquatic environment and must be quantified. The amount of the diet each fish consumed is also unknown; therefore, despite the effects observed, measuring the DOX concentration in larvae should be considered for further studies.

## 5. Conclusions

In conclusion, our data indicate that antioxidant supplementation effectively improves overall growth, increases mineralization, and rescues pro-oxidant-induced deformities in zebrafish. Antioxidants (RES, MT) may serve as a supplementation that can prevent and treat primary or secondary osteoporosis by counteracting pro-oxidant-induced ROS production and oxidative stress. Thus, the present study indicates the potential to introduce antioxidants as a candidate drug/supplement for studies in mammalian models to prove their potential use in osteoporosis treatment.

## Figures and Tables

**Table 1:** Sequences of primers used. All sequences in 5′–3′ orientation.

Gene	Primer Sequence		GenBank (Accession No.)
***oc2/bglapl***	Fw:	CCAACTCCGCATCAGACTCCGCATCA	NM_001291889
	Rev:	AGCAACACTCCGCTTCAGCAGCACAT	
***sp7***	Fw:	GCTAAGTCCAGGGCAGGCTCAG	NM_212863
	Rev:	CAATGGCGTGAAATCAGGAGTGTAAC	
***runx2b***	Fw:	TCAGGAATGCCTCAGGGGTTATG	NM_212862
	Rev:	CTTGCGGTGGGTTTGTGAATACT	
***eef1a1l1***	Fw:	TTGAGAAGAAAATCGGTGGTGCTG	NM_131263
	Rev:	GGAACGGTGTGATTGAGGGAAATTC	

## Data Availability

Not applicable.

## Supplementary Materials

The following supporting information can be downloaded at: https://www.mdpi.com/article/10.3390/nu14234959/s1, Figure S1: Comparison of the microdiets prepared with commercially available zebrafish fed (ZEBRAFEED) (**a**), Some of the skeletal deformities observed during the trial (**b**). Levels of significance were calculated using Tukey’s multiple comparisons (one-way ANOVA) [* *p* ≤ 0.05, ** *p* ≤ 0.01, *** *p* ≤ 0.001, **** *p* ≤ 0.0001].
