# Supplementary material for: Regular Supplementation with Antioxidants Rescues Doxorubicin-Induced Bone Deformities and Mineralization Delay in Zebrafish
Source: Nutrients. 2022 Nov 23;14(23):4959. doi: 10.3390/nu14234959 (PMC9739841; doi:10.3390/nu14234959)
Supplement: Supplementary file 1 [file nutrients-14-04959-s001.zip › nutrients-2045519-supplementary.pdf]

Supplementary Materials

Regular Supplementation with Antioxidants Rescues Doxorubicin-Induced Bone Deformities and Mineralization Delay in Zebrafish

Sunil Poudel [1,2,3], Gil Martins [1,2,3], M. Leonor Cancela [1,2,4] and Paulo J. Gavaia [1,2,*]

1 Centre of Marine Sciences, University of Algarve, 8005-139 Faro, Portugal
2 Faculty of Medicine and Biomedical Sciences (FMCB), University of Algarve, 8005-139 Faro, Portugal
3 PhD Program in Biomedical Sciences, Faculty of Medicine and Biomedical Sciences (FMCB), University of Algarve, 8005-139 Faro, Portugal
4 Algarve Biomedical Center, University of Algarve, 8005-139 Faro, Portugal
* Correspondence: pgavaia@ualg.pt; Tel.: +351-289-800-900 or +351-289-800-057 (ext. 7057); Fax: +351-289-800-069

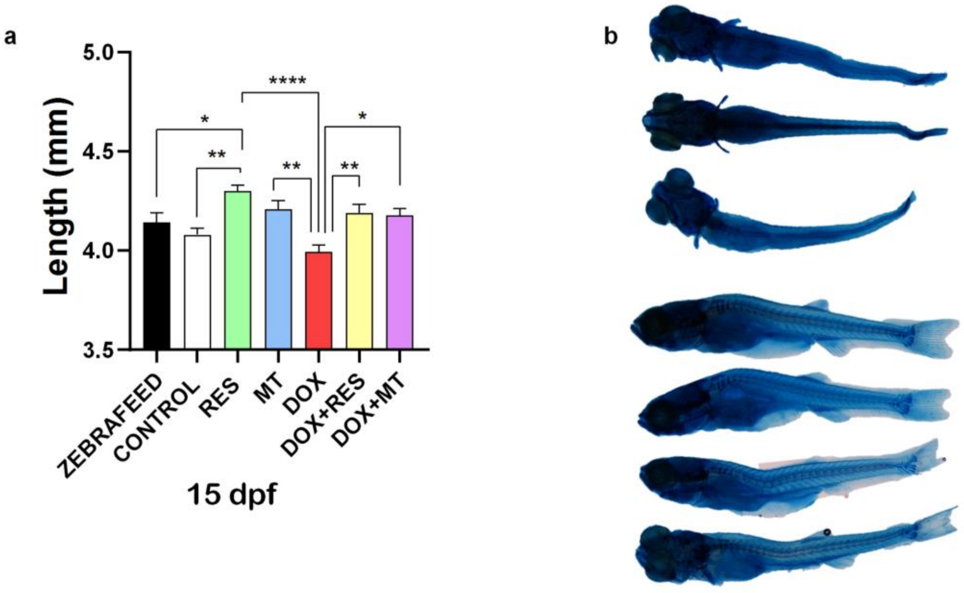

Figure S1: Comparison of the microdiets prepared with commercially available zebrafish fed (ZEBRAFEED) (**a**), Some of the skeletal deformities observed during the trial (**b**). Levels of significance were calculated using Tukey's multiple comparisons (one-way ANOVA) [* $p \leqslant 0.05$, ** $p \leqslant 0.01$, *** $p \leqslant 0.001$, **** $p \leqslant 0.0001$].
